# Letter to the Editor About the Study Titled ‘Early Mortality Predictors in Infective Endocarditis Patients: A Single-Center Surgical Experience’

**DOI:** 10.21470/1678-9741-2022-0419

**Published:** 2023-06-14

**Authors:** Bülend Ketenci, Fatih Kizilyel

**Affiliations:** 1 Department of Cardiovascular Surgery, Dr. Siyami Ersek Thoracic and Cardiovascular Surgery Training and Research Hospital, Istanbul, Turkey. E-mail: bulendketenci@gmail.com

We read the study titled “Early Mortality Predictors in Infective Endocarditis Patients: A Single-Center Surgical Experience” with interest. Infective endocarditis is a disease for which it is increasingly difficult to decide on diagnosis and treatment modalities because of its clinical course, causative microorganisms, and previous interventions (implantable device, transcatheter aortic valve implantation [TAVI]). Although antibiotic therapy and surgical methods have evolved in recent years, there has been no decrease in mortality. For this reason, it is recommended that infective endocarditis be evaluated by an *“endocarditis team”* that includes a cardiologist, a cardiac surgeon, an infectious disease specialist, and physicians from the field who intervene when complications may arise. This approach significantly reduces patient mortality^[1]^.

When heart failure develops due to infective endocarditis or when findings indicate that heart failure cannot be controlled despite antibiotic therapy^[2]^- such as abscess, pseudoaneurysm, enlargement of vegetations, and conduction disturbances like heart block -, the indication for surgery can be readily given. The timing of surgical intervention for infective endocarditis diagnosed after the occurrence of a neurologic event should be determined depending on the patient^[3]^. While the causative microorganism (fungus, resistant microorganisms), the size of the vegetation (> 10 mm)^[4]^, and the endocarditis of the prosthetic valve necessitate surgical intervention, it should not be overlooked that the initiation of antibiotic therapy reduces the possibility of embolism and surgery should not be rushed (waiting three weeks is recommended) in cases of bleeding-related neurologic events^[5]^.

Another significant issue is the presence of splenic abscess in a patient with infective endocarditis. It is clear that an untreated abscess after surgery for infective endocarditis is a source of reinfection. Therefore, it is critical to perform a splenectomy first, if possible, to prevent recurrence of endocarditis.

With the increase in TAVIs in recent years, infective endocarditis after TAVI has become one of the problems we have to deal with. The incidence of infective endocarditis after TAVI is more frequent than after biological valve replacement. TAVI endocarditis is a pathology with high mortality in patients who are considered high-risk for surgery.
